# Bioactive compounds, nutritional profile and health benefits of colostrum: a review

**DOI:** 10.1186/s43014-022-00104-1

**Published:** 2022-10-25

**Authors:** Amrita Poonia, Shiva

**Affiliations:** Department of Dairy Science and Food Technology, Institute of Agricultural Sciences, Banaras Hindu University, Varanasi, Uttar Pradesh 221005 India

**Keywords:** Colostrum, Functional foods, Bioactive compounds, Health benefits, Microbial infections

## Abstract

Bovine colostrum is defined as first milk by milching animals responsible for providing the innate immunity to the neonatal and possess many immunoglobulins for preventing the calf from diseases. Colostrum consist of many bioactive compounds like proteins, enzymes, growth factors, immunoglobulins and nucleotides that provides several benefits to human health. Numerous clinical and pre-clinical studies have demonstrated the therapeutic benefits of the bovine colostrum. This review focusses on bioactive compounds, their health benefits, potential of colostrum for developing several health foods and prevention of respiratory and gastrointestinal tract disorders. Processing can also be done to extend shelf-life and extraction of bioactive constituents either as encapsulated or as extracts. The products derived from bovine colostrum are high-end supplements possessing high nutraceutical value.

**Graphical Abstract:**

Processing of the BC to extend the shelf -life to obtain bioactive compounds for manufacturing functional foods.
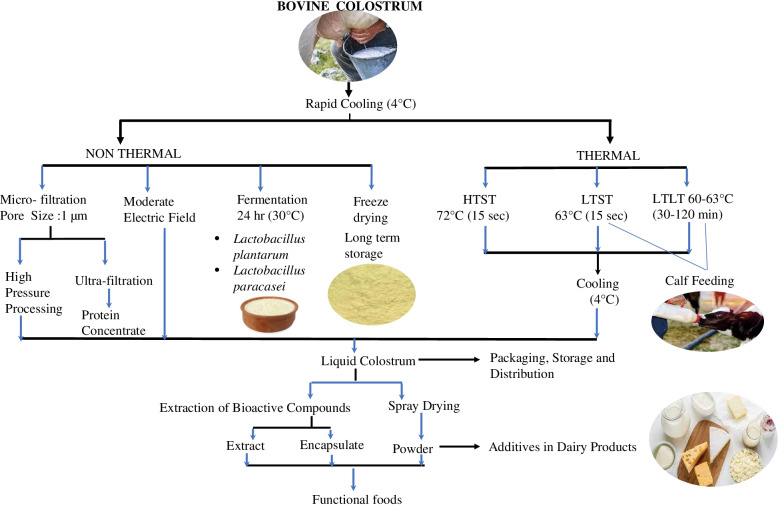

## Introduction

Bovine colostrum (BC) is defined as the first milk produced by the mammary glands of the milching animal after notably within first days (Rathe et al. 2014). Approximately about 5–10 L of colostrum can be produced by a healthy cow per milking (Moore et al. 2009). Due to the importance of colostrum, it is occasionally also referred as “Liquid Gold” (Kuralkar & Kuralkar 2010). Colostrum is profuse in bioactive compounds like immunoglobulins, growth factors, lysozymes, lectoferrin, lactoperoxidase and designed in such a way that it dispense the new born with passive immunity against pathogens. It also possesses elevated levels of fats, proteins, minerals and vitamins (Gomes et al. 2021). There are many evidences contributing to the verity that BC can be a valuable commodity for the treatment of diversified medical constraints in both adults and children (Bagwe-Parab et al. 2020; Ulfman et al. 2018). The components possessing immunomodulating nature are responsible for recovering and maintaining the health of gastrointestinal tract ultimately providing the useful outcomes for the treatment of gastro disorders and also on prophylaxis (Dzik et al. 2017). Colostrum can also be used as a potential heath supplements aiming in improvement of exercise performance and recovery of the athletes (Kotsis et al. 2018). So, the use of colostrum is not restricted to human beings but also for the well-being of the domestic pets and animals (Playford & Weiser 2021). There are clinical relevance cases and no contradictions showing the ill effects of intake of the higher dose of colostrum (Menchetti et al. 2016; Davison et al. 2020). Ceniti et al. (2022) reported that colostrum-based supplements may play a vital role in the prevention and treatment of various diseases as well as for pharmaceutical purposes or as a daily food supplement.

Due to higher protein content of BC along with lower temperature of clotting creates several problems during industrial processing and on contrary the antimicrobial activity drops the likelihood of fermentation (Tripathi & Vashishtha 2006). Most of the metabolites present in colostrum are gently affected or preserved well by pasteurization at 60 °C for 25 to 30 min (Xu et al. 2021 & Nguyen et al. 2019). The bioactive components of the colostrum are generally spray dried or freeze dried in order to maintain the biological activity and are made commercially available (Borad & Singh 2018) which are ready to consume with addition of whole milk, skimmed- milk and whey powder (Marnila & Korhonen 2011).

The diet of bovine plays a major role in the composition of colostrum (Coroian et al. 2013). Beside diet, the age of animal, health, lactation period, genetics, season and breed also plays a role in variation of the composition of bovine colostrum (Zarcula et al. 2010). The percentage of lactose is lower in the colostrum as compare to mature milk but the nucleotides, growth factors and cytokines get reduced after 3 days of parturition (Sacerdote et al. 2013). The amount of the colostrum produced by cow is enormous when juxtaposed with calf requirement (McGrath et al. 2016). This review aims to provide an extensive overview of the bioactive compounds of colostrum as a nutraceutical and food supplement.

## Nutritional profile of bovine colostrum

### Fat

The percentage of fat in colostrum is higher than the mature milk and the composition also distinctive (Arslan et al. 2021). Several changes are observed in the colostrum composition during its transition to mature milk and the concentration of palmitoleic, palmitic, and myristic acid is higher (O’Callaghan et al. 2020). Monounsaturated fatty acid in BC is approximately (24.0 to 28.0%), saturated (65.0 to 75.0%) and polyunsaturated (4.0 to 5.0%) and the principal fatty acids involves palmitic (40%) and oleic (21%), respectively (Contarini et al. 2014; O’Callaghan et al. 2020). There are lot of evidences convicting the palmitate importance in the early nutrition (Miles & Calder 2017) and immunomodulating benefits of oleic acid aiding in cardiovascular health (Sales-Campos et al. 2013). Some of research evidences support the fact about the role of fatty acids as molecules responsible for signaling and their contribution in the lipogenesis regulation in liver and also as dietary fats (German et al. 2019).

### Vitamins and minerals

BC consists of fat soluble vitamins like A, E, D, K and water soluble such as B-complex vitamins that plays important role in various metabolic mechanisms including antioxidant activity and the bone growth. Vitamin D has been involved in the support of immunity system and the mental health too (Cleminson et al. 2016). The vitamins concentration of colostrum is dependent on several factors. Fattah et al. (2012) studied that bovine colostrum contains 60 to 1040 μg.100 g-^1^ of vitamin E as compared to milk. BC is also rich in minerals like calcium, magnesium, copper, phosphorus, iron, manganese and zinc (Table 1).

**Table 1:** Comparison of composition of bovine colostrum with mature milk

Components	Mature Milk	Bovine Colostrum	References
Total solids (%)	12.9	24–28	Playford and Weiser (2021)
Fat (%)	3.6–4.0	6–7	Zou et al. (2015)
Palmitoleic ω-7 C16:1	1.73–1.75	1.57–1.58	
Linoleic ω-6 C18:2	7.06–7.09	6.53–6.55	
Linolenic ω-3 C18:3	0.43–0.45	0.61–0.63	
Oleic ω-9 trans C18:1	0.59–0.60	0.76–0.77	
Gadoleic ω-9 C20:1	0.75–0.85	1.02–1.05	
PUFA	9.45–9.55	10.28–11.00	
MUFA	36.69–37.10	44.01–44.05	
SFA	53.86–53.96	45.71–46.00	
Saturated Fatty Acid	53.86–53.90	45.71–45.90	
Lactose (%)	4.7–5.0	2.0–3.0	McGrath et al. (2016)
**Proteins**			
Protein (%)	3.1–3.2	14.0–16.0	Playford and Weiser (2021); Samarütel et al. (2016)
Albumin (%)	0.4–0.5	1.7–3.49	
Casein (%)	2.5–2.6	4.8–5.0	
**Immunoglobulins**			
Immunoglobulins (mg/mL)	0.4–0.9	42.0–90.0	Elfstrand et al. (2002); (Buttar et al. (2017); Bagwe et al. (2015)
IgA (g/L)	0.04–0.06	3.2–6.2	
IgG1 (g/L)	0.31–0.40	34.0–87.0	
IgG2 (g/L)	0.03–0.08	1.6–6.0	
IgM (g/L)	0.03–0.06	3.7–6.1	
**Minerals**			
Sodium (g/kg)	≈ 0.4	0.7–1.1	Godden et al. (2019)
Calcium (g/kg)	1.2–1.3	2.6–4.7	
Potassium (g/kg)	1.5–1.7	1.4–2.8	
Magnesium (g/kg)	0.11–0.13	0.4–0.7	
Phosphorus (g/kg)	0.9–1.2	≈ 4.5	
Zinc (mg/kg)	3.0–6.0	11.6–38.1	
**Vitamins**			
Vitamin A (μg/100 mL)	34.0–35.0	25.0–26.0	Arslan et al. (2021); Godden et al. (2019)
Tocopherol (E) (μg/g)	0.06	2.92–5.63	
Thiamin (B1) (μg/mL)	0.4–0.5	0.58–0.90	
Niacin (B3) (μg/mL)	0.8–0.9	0.34–0.96	
Riboflavin (B2) (μg/mL)	1.5–1.7	4.55–4.83	
Vitamin D (IU/g fat)	0.41	0.89–1.81	
**Antimicrobials**			
Lysozyme (mg/L)	0.07–0.6	0.14–0.7	Boudry et al. (2008)
Lactoferrin (g/L)	0.1–0.3	1.5–5.0	Boudry et al. (2008)
Lactoperoxidase (mg/L)	13.0–30.0	11.0–45.0	Arslan et al. (2021)
Growth hormone (mg/L)	< 0.03	< 1 mg	Bagwe-Parab et al. (2020)
Insulin-like growth factor-1 (μg/L)	200–600	50–2000	McGrath et al. (2016)
TGFβ1 (μg/L)	0.8–3.5	12.0–43.0	
Insulin (ng/mL)	0.042–0.34	4.2–34.4	Georgiev (2008)

### Carbohydrates

Lactose is predominant carbohydrate in BC, with concentration of about 2.50%. Lactose is found in lower concentration in BC when compared to matured milk of bovine or human. It can provide the glucose and galactose to the liver while supporting the storage and synthesis of glycogen (Playford & Weiser, 2021). A low level of lactose results in the production of milk that is extremely viscous and contains less water due to the absence of the osmoregulator lactose (Bleck et al. 2009; McGrath et al. 2016). Both glycans and oligosaccharides are present in bovine colostrum in acidic form ranging from 0.7 to 1.2 mg/mL which are less in matured milk. The main oligosaccharides are 6′ siayllactosamine, 3′ sialyllactose, disialyllactose and 6′ sialyllactose. Among all the above mentioned oligosaccharides the 3’siallactose contributes up- to 70% of total oligosaccharides (Arslan et al. 2021). The free oligosaccharides and conjugated N-glycans portray the greater number of prebiotic components (Karav et al. 2015). Several opportunities have been provided by the introduction of enzymatic glycosylation for the enhancement of the structure of bovine oligosaccharides to the human milk oligosaccharides (Weinborn et al. 2020). Although the experiments carried in pilot scale utilizing the bovine oligosaccharides have not yet showed much generalized changes in the gastrointestinal microbiota (Douëllou et al. 2017; Westreich et al. 2020).

The hybrid and complex glycans can be a source of prebiotics (Karav et al. 2016). Moreover, the conjugation of N-glycans with milk proteins allow them to be recovered by different methods. Further, the conjugation of these N-glycans to milk proteins enables different strategies for their recovery. Separation of protein from milk and followed by the treatment for the separation of N-glycans offer a potential avenue for their purifications (Bunyatratchata et al. 2020). Therefore, the derived N-glycans consisting the N-glycosylated proteins can be a formidable source of the substrates that are prebiotic in nature (Cao et al. 2019). Extensive characterization of N-glycans showed that they are extremely selective to particular bacteria in gut of adult. Further, the bacteria which are capable to access them are restricted due to relative huge repeated polymers having lesser complex nature of the oligosaccharides and thus contributing to limited selection by *Bifidobacterium* with improved GI barrier and declined enteric inflammations (Henrick et al. 2019; Duar et al. 2020).

Colostrum is considered as a liquid gold, immune milk and “superfood” due to its excellent nutritional profile, functional compounds and bioactive compounds when compared with single ingredients supplements. BC is very well known for its potential health benefits and some innovative technologies are required to explore novel methods to prepare colostrum enriched functional foods to enable their effective utilization in different food industries.

## Bioactive compounds of colostrum

**Fig. 1:**
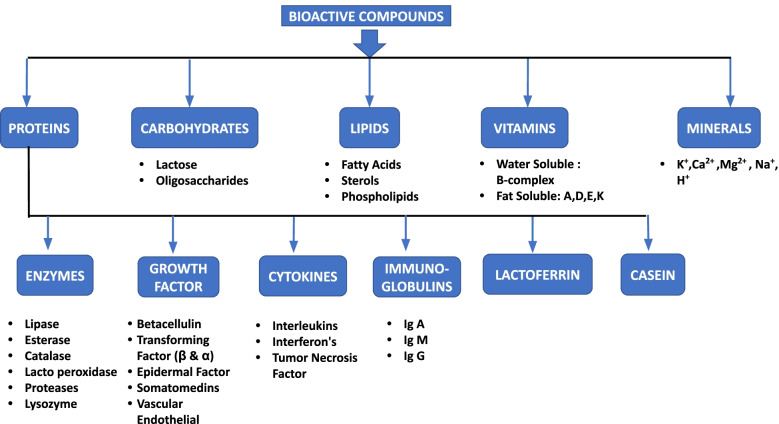
Overview of various bioactive compounds present in the bovine colostrum

There are about 250 or more functional compounds found in BC (Sienkiewicz et al. 2021). The colostrum have immnunomodulating components and contributes to direct as well as indirect mechanism for adjusting the immune response of host (Fasse et al. 2021). The food products prepared from cow colostrum contributes about 55.5% of global market in 2021 and the prognosis stipulated that there will be 6.4% increase per year of the colostrum products market from 2020 to 2030. This can be described by the spike in request and interest of several health promoting items, well associated with the health risks and illnesses owing from improper nutrition (Future Market Insights 2020). Bioactive compounds are present in higher amount in colostrum as compare to milk. Protein concentration in colostrum is on higher side during first 24 hours of post parturition (15.90 g/100 g) which decreased to (3.3 g /100 g) after 5 months of calving (Contarini et al. 2014). The immunoglobulin contributes about 70–80% of total protein and present (30 to 200 g/L) which get declined to (0.4to1.0 g/L) shortly after parturition (McGrath et al. 2016). The subclass namely IgG1 is responsible for accounting 75% antibody followed by other subcategories including IgM then IgA & IgG2 (Fasse et al. 2021). The bovine trypsin inhibitor is responsible for protecting the growth factors. It can also provide protection to the proteins having the biological activities and IgGs from proteolytic degradation taking place in the gut. The concentration of this trypsin inhibitor is 100 times more in the bovine colostrum, when compared to the normal matured milk (Godden et al. 2019). The concentration of α-lactalbumin is also high (1/4^th^of total whey protein) along with elevated amount of essential amino acids (Playford & Weiser 2021). Figure 1, depicted an overview of different types of bioactive compounds present in colostrum.

Saad et al. (2016) delineated that the bovine colostrum exhibit neutralizing effect for the endo-toxins and also show antimicrobial activity. Alsayed et al. (2020) reported that utilization of colostrum can lead the way to alteration in the mirobiome of the respiratory tract. So, the bioactive compounds present in colostrum can be beneficial to the athletes vulnerable to the infections of respiratory tract after physical trainings (Jones et al. 2016; Glówka et al. 2020). Bioactive compounds of BC isolated either from unvaccinated/vaccinated cows have nowadays scrutinized for their potentiality as a protecting agent in opposition to infections caused in humans by SARS-CoV-2 (COVID-19) (Mann & Ndung’u, 2020; Jawhara 2020).

### Immunoglobulins

These are related to the immunological activity and act as defense system for diseases and infections. Now-a-days, it is possible to formulate hyperimmune colostrum (HBC) preparation by the enrichment and fractionation techniques (Feeney et al. 2018). This sector is very interesting and industrial production must be focused owing of its good bioavailability (Anamika Das & Seth, 2017). IgG are found abundantly (80–90%) in colostrum with concentration ranging from 30 to 87 g L^− 1^ and lower amount of IgM, IgD, IgA and IgE (Godden et al. 2019). IgG can modulate the innate and adaptive immune responses and provides passive immunity. The immunoglobulins derived from colostrum are capable to fetch the pathogens with microphages, modify the microflora of intestine, stimulate the activation of B and T cell, and prevent the binding of pathogen to the host cell along with the localized production of IgA (Ulfman et al. 2018). Gasser et al. (2018) studied that the combination of polyvalent from of immunoglobulins with Vitamin D3 can beneficially impact on the systemic inflammation occurring in the patients suffering from the colorectal cancer by promoting the level of cytokines which have anti-inflammatory activity. The IgG is effective against the infections of gastrointestinal and respiratory tract like allergic asthma by their improved epithelial integrity as a barrier (Anderson et al. 2019). The concentration of IgA is higher in human in comparison to other immunoglobulins.

### Lactoferrin

It is multifunctional protein which belongs to the family of tranferrin and generally engaged in regulation of the iron. Lactoferrin is found in human breast milk and colostrum (Buttar et al. 2017). The average concentration of lactoferrin in cow colostrum is 0.2 g/L and 6 to 8 g/L. The health benefits owned by it includes antibacterial, anti-inflammatory activities and possess protection against varied gastrointestinal infections (Niaz et al. 2019). The N-terminal of lactoferrin interacts with the target and microbial hosts thus contribute to its biological activity (Legrand, 2016). The lactoferrin inhibit the infections caused by viruses by binding to the cells targeted and in turn obstructing the growth as well as intracellular replication of the viruses. The binding of lactoferrin to Heparan Sulfate Proteoglycans has also been shown in diversified cells (Sienkiewicz et al. 2021).

Research had also shown that it possess very good resistance to the digestion rate and provides the favoring benefit to the intestinal health of infant by reducing the microbial load (Manzoni, 2016). Wakabayashi et al. (2014) reported the inhibitory action of lactoferrin against viruses and the oral administration of lactoferrin showed favorable results and opposed to influenza herpes, common cold and viral gastroenteritis. It is capable enough to obstruct the growth of *L. monocytogenes* and *E. coli*. Lactoferrin in combination with Igs and Lactoperoxidase manifest synergistic effects (Fasse et al. 2021). Thus, this bioactive compound is an interesting sector of research for the scientists and researchers due to its multifunctional nature (Dzik et al. 2018).

### Enzymes

Enzymes are present in elevated amounts in post parturition periods in colostrum than matured milk. They play diversified role and their synergistic effect along with some proteins provide antimicrobial action. About 70 different indigenous enzymes have been recognized till now in the bovine milk with lysozymes and nucleases being the highest concentration in colostrum (Fasse et al. 2021).

### Lysozymes

This enzyme is bactericidal in nature and responsible for the destruction of cell wall of the bacteria (Ribeiro et al. 2016). Lysozymes hydrolyses the layer of peptidoglycan and leads to the lyses of both gram negative as well as gram positive counterparts. Lysozyme concentration in colostrum varies between 0.14 to 0.7 mg/L (Menchetti et al. 2016; McGrath et al. 2016). The average lysozyme concentration in colostrum is about 0.28 μg/mL and it also hydrolyzes bond which is β (1 → 4) present between N-acetylglucosamine & muramic acid. It is also effective against *Pseudomonas aeruginosa* and *E. coli* (Fasse et al. 2021).

### Lactoperoxidase

It is vital antimicrobial agent, commonly found in milk (Korhonen 2013). Lactoperoxidase is accountable in catalyzing the system having antimicrobial mechanism from the H_2_O_2_ and the SCN- (thiocyanate ion), eventually producing the reaction products which are capable enough to inhibit the growth of microorganisms such as fungi, bacteria and protozoa. The concentration of lactoperoxidase is lower in early post parturition days but gradually increases and become the enzyme with highest concentration in milk. It is normally engaged in natural defense of the body (Borad & Singh 2018; Menchetti et al. 2016). This enzyme is heat resistant, retards the bacterial metabolism and fabricated by the mammary gland of milking animal, and also in expression of the gene in epithelial cell for encoding of this particular enzyme. Additionally lactoperoxidase also safeguard the glands from several infections caused by many pathogenic microbes including *P. aeruginosa*, *Streptococcus* spp. (inflammatory infections) and *Listeria monocytogene*. This enzyme constitutes for about 1.25 to 2.5% of lactoperoxidase activity (Fasse et al. 2021). Korhonen (2013) reported the average activity of lactoperoxidase was 37.8 μg/mL in BC.

### Proteinases

The principal proteinase in milk is plasmin (serine protease). Its concentration is ten times higher than the mature milk. There is decrease in the activity of plasmin during the conversion of colostrum to mature milk. Along with this, the activity of Cathepsin D and the protease is lower in colostrum than the milk (McGrath et al. 2016).

### Role of micro RNA in bovine colostrum

MicroRNAs (miRNAs) are short, endogenous, noncoding RNA molecules of approximately 21–25 nucleotides (Lau et al. 2001). Higher amounts of miRNAs are present in colostrum than milk and immune and development related are more prominent. Etheridge et al. (2011) reported that MicroRNAs are very well known as biomarkers in human medicine for detection of many diseases such as breast cancer. MicroRNAs can play an important role in development of good diagnostic tools for the early detection of mastitis disease in animals as well as important indicators for the quality of different food products. MicroRNAs also includes the nutritive indexes of fluid milk and powdered formula milk (Chen et al. 2010). They are the main regulators for the different biological and developmental processes. In colostrum as well as in milk, miRNAs are the signaling molecules passed from mother to newborn. As they are packed in extracellular vesicles, which makes them resistant to harsh conditions in the gastrointestinal tract and reached in small intestine, absorbed and transferred into bloodstream. Van Hese et al. (2019) reported that miRNAs also regulates B and T-cell differentiation and affect interleukin production of macrophages.

## Growth factors of bovine colostrum

There are about 50 different polypeptides in the bovine colostrum termed as the growth factors. These perform multiple functions but their concentration is lower in the matured milk. Growth factors possess resistance for the heat treatment (up to 60 °C). Several newer technologies are being developed to extract such compounds from the bovine colostrum (Gomes et al. 2021). The concentration of growth factors varies with the species and also within the species. Some of the studies have reported the reduction of such factors in the bovine colostrum within the 48 to 72 hr. after calving (Playford & Weiser, 2021).

### β- transforming growth factor

The β-transforming growth factor (TGF-β1) is produced by various kinds of cells and responsible for regulating many cellular functions like oncogenesis, repression of the immune responses and proliferation of cells (Ihara et al. 2017). The structure of TGF-β1 is distinctive from that of TGF-α. They are largely articulated in gastrointestinal superficial zones. The TGF-β1has near about 35 isoforms. Beside this TGF-β also have anti-inflammatory and immunomodulatory activity and act as epithelial barrier of intestine (van Neerven 2014; Kelly et al. 2017). In addition to the epithelium, TGF-β can also modulate the immune cells and the luminal microbiota functions in the intestine, thus contributing to maintain intestinal homeostasis (Ihara et al. 2017). TGF-β engages in the suppression or inducement of immune responses which are adaptive in nature and in the regulation of innate immunity (Kelly et al. 2017). It can inhibit or control inflammation of the airway and the hyperactivity by T helper (effector). There are certain evidences contributing to the role of protein in the maintenance/development of immunity in the children. This can lead to ultimate protection of children form many inflammations and allergies (Batista da Silva Galdino et al. 2021).

The major growth factors involves β-cellulin (BTC), epidermal growth factor (EGF), β1-transforming growth factor (TGF-β1), insulin-like growth factor (IGF-1), fibroblast growth factor 1 & 2 (FGF1 & FGF2) and platelet-derived growth factor (PDGF) (McGrath et al. 2016). Polypeptides like TGF-β1, EGF and IGF-1 have the ability to be partially degraded or absorbed well intact in GI tract (Gomes et al. 2021).

### Cytokines

These compounds are exuded by the leukocytes, but some cytokines present in the colostrum are propagated in mammary glands of the bovine (Menchetti et al. 2016). Normally the cytokines like TNF-α, IL-1β and IL-6 are found in the bovine colostrum. Cytokines display anti or pro inflammatory action, and bracing the immunity against fungi, viruses and bacteria (Sienkiewicz et al. 2021). Cytokines are involved in stimulating the inflammation mechanism like IL-8 for the stimulation of the migration of cell lines of colonial cells. Furthermore, they play role in prevention of excess inflammation like cytokines IL-10 (Rathe et al. 2014; Saraiva et al. 2020).

### Leukocytes

The content of leukocytes in bovine colostrum is approximately about BC ∼ 106 /mL and fundamentally comprises the colostral mono-nuclear cells (CMCs) like lymphocytes and macrophages along with epithelial and polymorphonuclear cells. These are capable for modulating the responses of immune system and thus aids in maintenance of the equilibrium condition between tolerance of system and allergy (Sienkiewicz et al. 2021).

### Casein

The reported values of casein in colostrum and milk is approximately 9.24% and 2.5 to 2.8%, respectively. Casein-derived bioactive peptides exerts anti-oxidative, immunomodulatory activity and cytomodulatory effects, generated through enzymatic hydrolysis, fermentation, or gastrointestinal digestion (Fasse et al. 2021). The concentration of casein decreases after each milking among which the proportions of α_s_-casein reduced. There is increment in levels of к-casein, although the β-casein values remained constant (McGrath et al. 2016). The peptides of casein present opioid properties and Brooke-Taylor et al. (2017) studied that the β-casomorphin-7, μ-opioid peptide and some other short chain β-casomorphins (BCM-5, BCM-3 and BCM-4) are released via the digestion in GI form. Additionally, the hydrolysates prepared from both whey and casein have been attained to show interaction with receptors involved in responses of innate immunity (Kiewiet et al. 2017).

### Lactalbumin

The concentration of α-lactalbumin is higher in human milk as compare to bovine colostrum and matured milk. β-lactoglobulin is not found in human milk but abundantly present in bovine milk (Sienkiewicz et al. 2021). There is release of many peptides occur in the small intestine having the antimicrobial and the immunomoduatory activity released by the digestion of α-LA (Layman et al. 2018).

### Colostrinin

It is a complex rich in proline-peptides known as Proline Rich Polypeptides (PRP) found in colostrum of cow, goat, and human. The molecular weight of PRP range from 500 to 3000 Da (Janusz & ZabÅ, 2013). PRP discovered for their role in affecting the differentiation and maturation of thymocytes (murine) in the production of interleukin 6, the gamma-interferon (γ-IFN), factor of α - tumor necrosis (α -TNF) and some other types of cytokines. It was exhibited that some small constituent peptides of the PRP induce the augmentation of peripheral leukocytes in blood. It can reduce the stress in the cells due to oxidation and the cellular damage and suppresses signaling mediated by (4HNE)-4-hydroxynonenal (Zabłocka et al. 2020).

### Minor compounds of bovine colostrum using proteomic approach

Proteomics techniques have provided new approach to study milk proteins and main progress towards milk proteomics research. This technique is very helpful in studying the complex components of bovine colostrum and could have vital applications for care and management of neonate. Bovine colostrum proteome used centrifugation and ultracentrifugation for removal of fat layer and isolation of whey from casein. Immunoabsorption and 2-D gel electrophoresis were used to separate proteins before microsequencing and tandem MS analysis. Altomare et al. (2016) identified over 600 proteins that occur in low quantity in colostrum and also reported that ProteoMiner enrichment, gel separation, depletion, affinity chromatography and nano-HPLC-MS/MS techniques are the most expensive bovine colostrum proteome.

## Health benefits of bovine colostrum

The bioactive compounds of BC have potential for treating and preventing many gut and respiratory tract related diseases and malfunctions. Different forms of preserved colostrum used for human consumption includes as liquid, powder, tablets or capsules (Silva et al. 2019). Liquid BC is used as an ingredient in many dairy products such as cheese, dahi, *khees*, yoghurt, kefir, ice cream and milk based beverages. BC can be processed in many ways to extend the shelf -life and to obtain bioactive compounds for manufacturing functional foods.

### Respiratory tract

BC can prevent from the infections of the Upper-respiratory tract (URTIs) that affect the nose, trachea, mouth, larynx and throat, evoking laryngotracheitis, nasorafingitis, pharyngitis, sinusitis and laryngitis. There are evidences showing the oral supplementation of BC positively acts against URTIs among both the adults and children (Batista da Silva Galdino et al. 2021). Athletes, who are subjected to higher duration of exercise also increased chances of infections in respiratory tract. This might be because of the mild immunosuppression due to the prolonged overtraining and insufficient recovery of the body (Davison, 2013; Williams et al. 2019). The BC has been explored as the option for improving the immunity of the athletes (Gleeson & Pyne, 2016).

### Gastrointestinal tract disorders

Modulation of many immune responses takes place when diet is supplemented with BC. In recent years, research demonstrated the favorable effects against several disorders of the gastro intestine chiefly the inflammation, prevention/ treatment of secondary ulceration and viral diarrhea. Apparently, the BC anti-inflammatory activity on the epithelial cells of intestine is related to the repression of NF-κB (Nuclear factor involved in the immune and inflammatory responses). The BC also have the potential to be utilized in adjunctive therapy for easing the side effects induced by drug, among which the GI tract is weakened because of pharmacological therapies. BC is capable for the reduction of the oral and intestinal mucosa inflammation caused due to the cancer treatments (Gomes et al. 2021). Anderson et al. (2019) carried an experiment of cell culture with 4 processed products of colostrum and exhibited that industrially processed products have the potential to improve barrier integrity of small intestine. In another study the researchers investigated the BC administration effect on the permeability of intestine of 16 fighters (mixed martial arts) in the competitive season. The reports showed that there was restoration in less than 3 weeks of mild supplementation in the intestinal permeability (Halasa et al. 2017).

### Short bowel syndrome

Short Bowel Syndrome is the insufficiency of the intestine because of intestinal enucleation due to the infarction of mesenteric vessels, abnormalities, trauma or the Crohn’s disease compilations. It is identified by lower absorption of nutrients and diarrhea and onset of weight-loss and malnutrition (Jin & Gramlich, 2019). Gomes et al. (2021) studied the effect of supplementation of piglets with protein concentrate obtained from BC and promising results were derived such as improved levels of IGF-1, muscle hypertrophy and the binding proteins. So, supplementation of protein concentrate of BC can be alternative in treatment and prevention of Short Bowl Syndrome. However there was no persistent evidence of adaption of small bowl when feed with IGF-1 enriched BC diet. Alongside, stool normalization and usual weight gain in the test animals when fed with protein concentrate of BC was observed. The supplementation with protein concentrate of BC improved the intestinal morphological features like increased crypt depth and villus length leading to the intestinal adaptation (Gomes et al. 2021).

### Inflammatory bowel syndrome

It affects the intestine and is grouped into 2 phenotypes namely: Crohn’s disease and ulcerative colitis. The cause is not yet well clarified by the scientist but some studies suggested that this might be because of dis-functioning of mucosal immune system. Therefore, BC can be a substitute to supply the growth factors and the immune factors, leading to the recovery of GIT (Hisamatsu et al. 2013). An investigation related to the antibacterial and the anti-inflammatory effects of BC on the cells of intestine showed positive benefits on the intestine health (Chae et al. 2017). Menchetti et al. (2021) observed the modulation of microbiota of intestine and depletion of the inflammatory markers when evaluated for 7 days BC administration like a prophylactic measure in lessening colitis in mice. The stimulation of the growth of beneficial colonial microbes like *Lactobacillus* spp. and *Bifidobacterium* spp., was reported.

### Necrotizing enterocolitis

Necrotizing enterocolitis is a usual morbidity related to preterm birth and a principal cause of mortality of infants (Niño et al. 2016). Diversified studies have been conducted for the examination of the impacts of BC and human colostrum on outcomes of NEC and also in the development of the preterm. In a clinical trial conducted by Balachandran et al. (2017) 86 lower birth weight infants (1000 to 1500 g), the BC supplementation was provided in 2 g dose for 4 times in a day. No noteworthy differences were shown for the NEC occurrence when supplemented with BC in comparison to the placebo. Among the meta-analysis by Sadeghirad et al. (2018) cumulative findings suggested that neither BC nor human colostrum (HC) had affected the prevalence of NEC that is severe, feed intolerance, mortality, sepsis proved by culture.

### Microbial infections

BC is able to lessen the growth of bacteria including *Staphylococcus aureus*, *Salmonella typhi*, *Proteus vulgaris*, *Escherichia coli* and *Enterobacter aerogenes*. Many promising food products are developed and promoted that can probably be utilized like functional foods aiding in forestall of GI infections (Bartkiene et al. 2018). The authors examined the fermentation of BC with *Lactobacillus paracasei* and *Lactobacillues plantarum*, and effect of drying methods on Igs like IgM, IgG and IgA and its antimicrobial activity. The results depicted that product obtained from the colostrum fermentation inhibited 12 pathogenic microbes growth and also possessed higher IgM and IgG content (Bartkiene et al. 2020).

*H. pylori* is contemplated among most common infections in human by bacteria. The infection is caused by oral ingestion of the bacterium, transmitted in early childhood and its elimination in children can be spontaneous (Gomes et al. 2021). *H. pylori* infection is highly associated with several gastrointestinal disorders, including chronic gastritis, peptic and duodenal ulcers, and gastric adenocarcinoma (Kao et al. 2016). The addition of lactoferrin form BC aids in improvement of eradication rate of *H. pylori* infection (Table 2).

**Table 2:** Studies contributing to the health benefits of colostrum (Since 2016 onwards)

Disease	Form of colostrum	Administration mode	Inferences	References
Gastrointestinal carriage	Anti-UPEC Hyperimmune Bovine Colostrum (HBC)	De-fatted, concentrated, and freeze dried colostrum (suspended at 10% (w/v) in dH_2_O)	Disruption of colonization of ST131 UPEC in the gastrointestinal tract due to therapeutic value of HBC was observed when fed with IgG antibody purified from colostrum using a protein G column (GE Healthcare) as per the manufacturer’s instructions	Larcombe et al. (2019)
Respiratory tract infections (URTI or diarrhea)	Colostrum (liquid form)	6.0 g/day	BC supplementation have the tendency to suppress the inflammation of gut and stimulate mucosal integrity and tissue repair under different conditions related to tissue injury	Saad et al. (2016)
H1N1 (influenza A)	Colostrum (liquid form)	1.0 g per kg body weight	BC dose of 1.0 g per kg body weight showed the increased activity of natural killer cells	Wong et al. (2014)
Gut-barrier malfunction	Colostrum powder	20 g of BC thrice per day for 10 days	Reduction of intestinal permeability and incidence of diarrhea was reported	Eslamian et al. (2019)
Gut-barrier malfunction	Bovine colostrum powder	500 mg of BC twice a day for 20 days	Exhibited decrease of intestinal permeability	Halasa et al. (2017)
Infections (Acute diarrhoea) caused by Rotavirus and *E. coli*	Bovine colostrum powder	3.0 g of BC diluted in (50 ml of water) per day for 7 days	Displayed decrease in frequency as well as duration of vomiting and diarrhoea	Barakat et al. (2020)
Necrotizing enterocolitis	Bovine colostrum	BC mixed with human milk, 24 to 40 ml/kg/day	Exhibited improvement of gut function, bacterial defense mechanisms, nutrient absorption in comparison to formula-based fortifiers	Sun et al. (2019)
Necrotizing enterocolitis	Bovine colostrum		Reduction of feeding intolerance, NEC, delay-onset sepsis and mortality	Ismail et al. (2021)
Drug-induced lesions	Bovine colostrum (commercial products)	Commercially available bovine colostrum products, 2 mL (7 mg/mL) (orally administration)	Indomethacin damage (20 mg/kg, subcutaneously) was induced 30 min after gavage. Results depicted that there is restoration of gastric mucosa from the supplementation of commercially available BC products in male rats	Playford et al. (2020)
Drug-induced lesions	Spray-dried BC powder (Biofiber-Damino™)	7.5–30.0 g per day (thrice a day)	Decreased the peak severity of oral mucositis	Rathe et al. (2020)
Inflammatory bowel disease (Ulcerative colitis and Crohn’s disease)	Bovine colostrum	100–500 mg/kg/day IMM-124E (colostrum-based product)	100–500 mg/kg/day IMM-124E (colostrum-based product from cows, immunized with lipopolysaccharide (LPS) from *E. coli*) have the ability to decrease the levels of infiltrating immune cells and less pronounced mucosal damage Increase of regulatory T cells and serum levels of LPS binding protein	Spalinger et al. (2019)
Acute infectious diarrhea	Colostrum powder	3.0 g BC sachet with 50 ml water	Reported to lower the frequency of diarrhea and vomiting	Menchetti et al. (2016); Barakat et al. (2020)
Inflammatory bowel disease	BC powder	BC powder dissolved in saline solution (100 mg in 0.6 ml) for 1 week	Depicted that there was modulation of pro-inflammatory cytokines, TLR4 and microbiota	Menchetti et al. (2020)
*Helicobacter pylori* infection	Lactoferrin derived from BC	200 mg (Pravotin™), taken after 2 h of break-fast and 100 mg after 2 h of dinner for 1 week	Improvement of the eradication rate of *H. pylori* was observed	Megahed et al. (2017)
Gut-barrier malfunction	BC powder	20 g of BC/ day, 3 times per day, for 10 days, enteral nutrition (ICU Patient)	Decrease of intestinal permeability and occurrence of diarrhea	Eslamian et al. (2019)
TNBS- induced colitis.	Nutra-Summa Pure BC Powder®	100 mg of colostrum dissolved in 0.6 mL of saline solution for 21 days	*Enterococci* and *E.coli* count was increased but *Bifidobacterium*, anaerobes and *lactobacilli* reduced	Filipescu et al. (2018)
Antimicrobial activity	BC fortifier Powder	2.8 g of BC Fortifier mixed in 100 ml of human milk	The results depicted that the physico-chemical properties were modified along with the prevention of in-vitro bacteria responsible in the neonatal sepsis	Gao et al. (2021)
**Human studies**				
Respiratory tract	Bovine colostrum	20 g of BC mixed with 250-300 ml of water and twice a day	Can cut down the decrease in immunity in cases of the athletes performing prolonged exercises	Jones et al. (2019)
Intestinal permeability	Freeze dried BC	500 mg of BC was given twice a day for 20 days	The test performed was mannitol/lactulose (M/L) test which is differential sugar absorption. The BC collected after 2 & 24 hrs resulted in significant reduction in permeability of the intestine of 31 athletes.	Hałasa et al. (2020)
Intestinal	BC powder	Supplemented with BC consisting of 35.3 mg IgA, 350 mg IgG & 25.3 mg mixed in 50 ml of water, given for 1 week.	160 children suffering from acute diarrhea were fed and after 48 hr., results of the study revealed that BC supplementation lowered the vomiting and diarrhea frequency and also was effective in the treatment of rotavirus and bacterial (*E. coli*) related diarrhea	Barakat et al. (2020)
HIV-related diarrhea	ColoPlus powder	50 g of BC based product possessing 3–4 g of IgG in 50 g, two times a day.	Open-labeled study was conducted on 30 subjects provided with the mentioned dose and Results suggested that products based on BC reduced diarrhea associated with HIV & the daily no. of bowel evacuations and also increased the CD4^+^ cell, hemoglobulin & albumin concentrations.	Florén et al. (2006)
Upper respiratory tract infection (URTI)	BC capsules	2 capsules of BC (500 mg) for 4 weeks	This supplementation was proved effective in the treatment of URTI nasal swab micro-biome in 27-year-old non-alcoholic, non-smoker patient.	Alsayed et al. (2020)
Respiratory allergies	BC	1000 mg BC for 3 months	A total of 19 children (7-18 years) suffering from respiratory tract allergies were supplemented with BC and reported with improved the nasal congestion & lungs functioning among the mono-sensitized groups suffering from allergies of respiratory tract.	Oloroso-Chavez et al. (2017)
Vulvovaginal atrophy	Gel	Applied 5 ml of gel (Monurelle Biogel) for 12 weeks	BC based purified gel given to 172 women suffering from vulvovaginal atrophy. They applied 5 ml of gel for 12 weeks after intercourse or intimate cleaning. The outcomes showed the potential of BC-based gel in the vulvovaginal atrophy treatment, improvement of sexual life.	Schiavi et al. (2019)
COVID-19 infection	Nutritional syrup	32 mg BC liposomal lactoferrin per 10 ml along with 12 mg of vitamin C and 4–6 doses for 10 days	The outcomes proved that this supplementation was effective in the prevention of COVID-19 infected 75 patients	Serrano et al. (2020)
NEC	BC concentrate	BC concentrate for 2 weeks	A randomized, double-blinded, placebo study was conducted in 80 preterm neonates supplemented with BC concentrate for 2 weeks. The outcomes showed that the hypo-caloric feeding of BC reduced the risk of NEC, morality and feeding intolerance.	Awad et al. (2020)
Skin disease	Lyophilized BC	Lyophilized BC (collected within 1 h after parturition) given @ 0.05–0.5% for a period of 24–48 hrs.	The results observed were found beneficial for the treatment of keratinocytes or skin diseases and for the cutaneous dryness among the old age people.	Kovacs et al. (2020)
Gastrointestinal disorders	Bovine colostrum product	0.3 g twice/day supplementation of BC oligosaccharides with bacteria named *Bifidobacterium longum* subspecies *Infanti* for 12 weeks	This dose was given to the children (2–11 years) suffering from the GI symptoms & autism disorders. A reduction in IL-13 and TNF-α production was noticed.	Sanctuary et al. (2019)

### Infectious diarrhea

This is a disorder of digestive tract and chiefly caused by pathogens like *E. coli*, *Cryptosporidium* spp., rotavirus and BC supplementation showed its action against such pathogens. Over past years rich evidences supporting the fact that BC can prevent viral diarrhea has been congregated (Bagwe et al. 2015). The BC role in diarrhea infections with randomized and controlled trails, meta-analysis and systematic search depicted that the products made from BC lighten the infectious diarrheal symptoms among children (Li et al. 2019). BC is capable to protect the neonatal calf form pathogens, like Enterotoxigenic *E.coli* (ETEC) which is the primary cause of neonatal diarrhea in calf by providing passive immunity. Kolenda et al. (2015) conducted a clinical trial for examination of the difference among BC and HBC on the children suffering from shigellosis caused by *Shigella dysenteriae.* No improvement was found in patients suffering from shigellosis when compared to antibiotic therapy. All these findings show the ability of HBC on the *S. dysenteriae* progression which invades the epithelial cells (Arslan et al. 2021).

### Viral influenza

Viral influenza is accountable for respiratory infections and Type A & Type B genera that affects the humans with the symptoms like mild infection in the upper respiratory area, coughing, sore throat, headache, fever, muscle pain, runny nose and fatigue to elevated conditions and leading to severe pneumonia. The mode of transmission is via air in aerosol or droplets of the respiratory secretions form infected person to another person (Krammer et al. 2018). The oral administration of BC can be helpful in treatment or prevention of the influenza and sometimes can be equally successful against the infection compared to the standard interventions (Batista da Silva Galdino et al. 2021).

### Assist in therapy for management of COVID-19

BC contains lactoferrin as a bioactive compounds which helps to slow down the disease progression. BC plays dual role as an antibacterial, anti-inflammatory and anti-viral as well as also strengthen the human immune system. Yadav et al. (2016) reported that bovine colostrum has strong antimicrobial activity against Gram negative and Gram positive strains of microorganisms. Colostrum also exhibits 100 μg/ml minimal inhibitory concentration (MIC) against *S. aureus*, *P. vulgaris*, *E. coli* and *S. typhi*. All these facts formulate a base that it is possible to state that it might have viricidal effects against COVID-19 virus. Lactyferrin Forte™ (oral supplement with lactoferrin) was given to 75 COVID-19 positive patients for 10 days and found that there was a reduction in headache, dry cough and diarrhea and an improvement in muscle pain shortness of breath and anosmia (Serrano et al. 2020). In another study Serrano et al. (2020) studied that 128–192 mg/day of the supplement was given to 256 peoples who had contacted with the COVID-19 positive patients. The authors reported a preventive role of the supplement against infection by the virus and lactoferrin seems to be effective in prevention as well as treatment of people affected by COVID-19.

## Key differences between bovine colostrum and human colostrum

Human colostrum (HC) performs peculiar part in the support of newborn as a protective agent against many chronic diseases and allergies, along with providing metabolic benefits for long time. The peptides in human milk manifest different functions, including antibacterial, mineral binding, immunomodulatory, antioxidant, antihypertensive activity, antithrombotic, and the opioid antagonism effect (Bardanzellu et al. 2017). Both the HC and BC are rich source of protein, growth factors, immunoglobulin, lactoferrin and many other bioactive compounds which are capable to treat autoimmune disorders (Godhia & Patel 2013). The HC contains high amount of whey as compared to the matured milk (Juhl 2017). The concentrations of bioactive compounds like EGF, IGF, lactoferrin, growth hormone and IgA are noticeably higher in case of HC as compared to BC (Bagwe et al. 2015; Godhia & Patel 2013). Whereas cow colostrum have 20% higher content of IgG than HC having about 2% IgG (Thapa 2005). Comparison of the BC and HC composition is mentioned in Table 3.

**Table 3:** Comparison of bovine colostrum with human colostrum

Components	Bovine colostrum	Human colostrum	References
Energy (kcal)	~ 130	58	Kehoe et al. (2007)
Fat	~ 6.7	3–5%	Guthrie (1989)
Protein(g/100 ml)	~ 14.9	0.8–0.9	Bagwe et al. (2015)
Lactose(g/100 ml)	~ 2.6	6.9–7.2	Buttar et al. (2017)
**Immune factors**			
Lactoferrin (mg/ml)	100.0	700.0	Stelwagen et al. (2009)
IgM (mg/ml)	3.7–6.1	1.59	
IgG (mg/ml)	34.0–87.0	0.43	
IgG2 (mg/ml)	1.6–6.0	–	
IgA (mg/ml)	3.2–6.2	17.35	
**Growth Factors**			
Growth hormone (GH) ng/L	≤ 0.03	41.0	Playford et al. (2000); Kaducu et al. (2011)
Insulin like growth factor (IGF) mg/L	10	18.0	
Epidermal growth factor (EGF) mcg/L	30–50	200.0	
Vascular endothelial growth factor (VEGF) mcg/L	NA	75.0	
Transforming growth factor (TGF α) mcg/L	2.2–7.2	2.2–7.2	
TGF β mg/L	1–2	20–40	

Research has exhibited that the BC have 100 to 1000-fold more potential than the HC. So, human infants can also thrive well on consuming the infant formulas based on BC. As it can provide the infant with growth factors and passive immunity required for the GI as well as physical development (Bagwe-Parab et al. 2020). Liang et al. (2018) reported that the total content of free amino acid in BC & HC was 0.32 g L^− 1^ & 0.63 g L^− 1^ which is twice in HC than that of BC. Nevertheless, the total content of hydrolytic amino acid was 4.2 g L^− 1^ & 2.2 g L^− 1^ in BC and HC, respectively. A grand total of 920 exosomes of milk proteins were quantified and identified in BC & HC by (Yang et al. 2017).

## Commercially available bovine colostrum supplements

There are many BC supplements manufactured worldwide, available in various forms such as capsule, tablets, gels and powder. Some of the supplements are Biostrum/B-colostrum manufactured by Nutritech Pvt. Ltd. in the form of capsule containing components like IgD, IgE, IgA, IgM and IgE with the serving size ranging from 4 to 6 capsule twice per day. Symbiotics colostrum powder having BC Phospholipids with serving size of 3.0 g. Zenith Nutrition Colostrum in the form of capsule containing IgD, IgE, IgA, IgM and IgE whose serving size is 2 capsule (Godhia & Patel, 2013). APS BioGroup, Mt Capra Wholefood Nutritionals, Biostrum Nutritech Private Ltd., are some of the company’s manufacturing colostrum powder form cow, goat and buffalo colostrum (Bagwe et al. 2015).

Other commercially available products based on the BC and its derivatives are Igazym lozenges consisting of lysozyme and powdered colostrum manufactured by Pedersen Biotech in Vejle Denmark; Smart Naco, SDN, Malaysia manufacturing product named Naco IgG Plus which is skimmed BC; Emma cream based on colostrum & Extra Edge BC powder by Immuno-Dynamics Inc. in Fennimore; Lactobin IG enriched spray dried powdered product by Biotest Pharma, Germany; ColoPlus by ColoPlus^AB^ in Sweden (Rathe et al. 2014). Novatreat Pvt. Ltd. in Turku, Finland produced world’s first drinkable product based on BC (Mehra et al. 2006).

### Overcoming hurdles during commercialization of colostrum

The microflora and nutritional profile of raw bovine colostrum is likely to be highly diverse and also increased possibility for the growth spoilage and pathogenic microorganisms. Consumption of raw bovine colostrum increases the chances of illness owing to spoilage, intoxication and infections. So, there is a need of imparting different types of heat treatments to bovine colostrum before consumption. There are various other hurdles faced during processing of colostrum for commercial purposes.

### Contamination by bovine pathogens

Many pathogens including *Salmonella* Spp., *L. monocytogenes*, *Mycobacterium bovis*, *Mycoplasma* spp., *Mycobacterium avium* subsp. *Paratuberculosis, Compylobacter* spp., and *E. coli* may be transmitted to dairy calves via colostrum. Heat treatment such as pasteurization and sterilization and Ultra High Temperature (UHT) can be used for destructions of all these pathogens. Use of different combinations of heat treatments also reduces the Igs concentrations and increases the viscosity of bovine colostrum.

### Control-on-farm constraints

The collection and storage of colostrum are the major factors in determining the shelf life of the colostrum during primary production (Phipps et al. 2016). Udder of the animal, environmental conditions, workers and contaminated equipment’s are primary source of contamination. Microbes may contaminate bovine colostrum due to poor hygienic conditions, milking, processing, packaging, transport and storage. According to European Union regulations, bovine colostrum must immediately be cooled below 8 °C to evade the fast accumulation of high bacterial counts and stored separately and temperature must be kept below 10 °C during transportation.

### Control of risks in processing bovine colostrum

The industrial processing of bovine colostrum is an urgent need to avoid the potential pathogens being inherent in unprocessed milk and milk products. Food Standards Australia New Zealand (2019, 2020) reported that health condition of animal, milking hygiene, chilling facility and efficiency, cleaning practices of equipment’s, maintenance and disinfection, personal hygiene of the workers and chilling temperature during packaging, storage and transportation are some important steps for maintaining the quality of BC prior and during processing. Several processing techniques used to destroy pathogens and improve the quality of colostrum includes Low Temperature Short Time (LTST) and High Temperature Short Time (HTST) pasteurization, Ultra-Heat Temperature (UHT) High Pressure Processing (HPP), microfiltration and fermentation. Nanotechnology and liposomal technology are some advanced technologies used to process BC for human consumption with many advantages.

### Processing challenges

Preservation of colostrum and its prevention from microbial contamination is one the main challenge for processing and development of colostrum based products for human consumption. Various problems occurring during industrial processes are the low clotting temperature and high protein content of BC which interferes with heat treatments such as pasteurization. High content of antimicrobial compounds in colostrum may inhibit or slow down the fermentation process (McMartin et al. 2006). Bioactive compounds including Igs, with nutraceutical value for humans are degrading by using high temperature for bovine colostrum. Separation of bioactive fractions from colostrum remains a huge challenge to both study the mechanisms by which this fluid can act on humans as well as for product development.

### Loss of nutrients and bioactive compounds

Bovine colostrum contains many bioactive compounds that needs to be preserved during processing. However, since most bioactive compounds are fragile, their stabilization is a big challenge for many applications. Heat treatment is the most common to ensure the microbial safety but adversely affect the bioactive compounds. Some recent technologies including freeze drying, high pressure and irradiation are recommended for enhancing shelf-life of bovine colostrum (Borad & Singh 2018). The authors reported that high pressure processing is a promising method for preservation of Igs. Tacoma et al. (2017) studied the effect of heat treatment on the proteome of colostrum identified by LC-MS/MS and reported an altered composition of high and low abundance proteins. Nanoencapsulation technology encapsulates the bioactive compounds present in colostrum into nanometer sized vesicles for prevention of their denaturation (Srinivas et al. 2010). This technology is very efficient to avoid instability problems during preservation of BC and its bioactive proteins. Chen et al. (1999) studied the encapsulation of IgG by using 0.5% of Tween 80, soy protein, sucrose stearate and reported enhanced stability of free IgG against pH 12.0 and 2.0 environments by 33–62% and 21–56%, respectively.

### Regulatory issue of bovine colostrum for commercialization

Bovine colostrum has been used since domestication of the animals and thousand years back as a human food in India. In United Stated, it is used as an antibacterial agent until the development of antibiotics. On the Western World, bovine colostrum is used in the form of medicine for boosting the immune system since eighteenth century (Godhia & Patel 2013). Regulation of the Industrial and Sanitary Inspection of Products of Animal Origin has prohibited the commercialization of bovine colostrum for human consumption in Brazil (Brasil, Ministério da Agricultura, Pecuária e Abastecimento – MAPA 2017). The US Food and Drug Administration (FDA) has recognized the safety of hyperimmune milks on the basis that no adverse health effects have been shown in clinical studies. Lactoferrin extracted from colostrum and added to conventional formulas in some countries and considered as safe food supplement by FDA. Regulation (EC) 853/2004 states that raw milk and colostrum must have a SPC at 30 °C of ≤10^5^ CFU/mL (Rathe et al. 2014). Bovine colostrum shall fulfil the provisions of the Food Safety and Standards (Contaminants, Toxins, and Residues) Regulations, (2011). It should be prepared and handled according the requirements specified in Schedule 4 of the FSSAI (Licensing and Registration of Food Businesses) regulations 2011 and other guidelines as may be specified from time to time under the FSSAI act 2006.

## Conclusions

The bovine colostrum own a distinctive combination of bioactive compounds and nutrients, ultimately making it a good natural source of high valued molecules. These bioactive compounds can be used for the manufacturing of high-end functional foods products. There are variety of bioactive constituents in BC ranging from immunoglobulins, enzymes to growth factors. BC is a promising and nutritionally attractive source due to its high value proteins and less lactose content. Although in India, the history of utilization of bovine colostrum goes back to thousand years but its use as a supplement is quite recent. The use of BC as a supplement is slowly and steadily gaining interest of the researchers and the consumers because of its respective health benefits. There are many studies providing a sound proof for the beneficial impacts of the bovine colostrum in treatment of various ailments as well as the prevention of many respiratory and gastrointestinal related issues like Short Bowl syndrome, Infectious diarrhea, Travelers’ diarrhea, and Necrotizing enterocolitis. Furthermore, BC has the potential to enhance the respiratory ability of the athletes.

The processing conducted on-farm can improve the BC quality and also extend its shelf life. New and non- thermal technologies such as High Pressure Processing, Micro-filtration and Moderate Electric filed has been used as a pasteurization alternative which can prevent the destruction of bioactive compounds occurring due to heat treatment. Nevertheless, these newer technologies are not very cost effective and can lead to increment of cost of the end product. Future placebo and clinical trials are required in order to address the gaps in knowledge of BC mechanism in GI disorders and weather the fractionated or whole BC is good for human consumption.

## Data Availability

The datasets used and/or analyzed during the current study are available from the corresponding author on request.

## Abbreviations

NEC: Necrotizing enterocolitis
BC: Bovine colostrum
URTIs: Upper-respiratory tract
HBC: Hyperimmune bovine colostrum
TGF: Transforming growth factor
